# Aneuploidy Patterns and Chaotic Embryos in IVF: Age-Stratified Analysis and Re-Biopsy Outcomes from a Romanian Cohort

**DOI:** 10.3390/medicina62020247

**Published:** 2026-01-24

**Authors:** Anca Huniadi, Petronela Naghi, Iona Zaha, Adelin Marcu, Liana Stefan, Liliana Sachelarie, Ioana Cristina Rotar

**Affiliations:** 1 Faculty of Medicine and Pharmacy, University of Oradea, 1st December Square 10, 410073 Oradea, Romania; ahuniadi@uoradea.ro (A.H.); lantal@uoradea.ro (L.S.); 2 Calla—Infertility Diagnostic and Treatment Center, Constantin A. Rosetti Street, 410103 Oradea, Romania; petronelanaghi@gmail.com (P.N.); adelinmarcu890@yahoo.com (A.M.); 3 Department of Clinical Discipline, Apollonia University, 700511 Iasi, Romania; 4 Faculty of Medicine and Pharmacy, Iuliu Hatieganu University, 400347 Cluj-Napoca, Romania; cristina.rotar@umfcluj.ro

**Keywords:** aneuploidy, chaotic embryos, mosaicism, PGT-A, maternal age, embryo quality, IVF

## Abstract

*Background and Objectives*: Aneuploidy is the leading cause of implantation failure and miscarriage, with prevalence increasing with maternal age. Embryos classified as chaotic, characterized by the presence of five or more chromosomal abnormalities, and those with complex aneuploidies, defined by two to four abnormalities, represent a controversial category in preimplantation genetic testing for aneuploidy (PGT-A), as the potential for misclassification remains a significant concern. *Materials and Methods*: We performed a retrospective study at the Calla IVF Center, Oradea, analyzing 230 blastocysts grouped by maternal age (25–30, 31–35, 36–40, and 41–50 years). A trophoblast biopsy was performed on days 5–7, and the samples were analyzed by next-generation sequencing (NGS). Embryos were classified as euploid, aneuploid, mosaic, or chaotic. The 19 embryos initially diagnosed as chaotic were thawed and subjected to re-biopsy. Statistical analysis included descriptive statistics (chi-square tests and ANOVA) and multivariable regression models, with *p* < 0.05 as the criterion for statistical significance. *Results*: Aneuploidy increased with maternal age, from 29.6% in women aged 25–30 years to 68.7% in those aged 41–50 (*p* = 0.002). Poor-quality blastocysts exhibited higher aneuploidy rates (72.4%) than good-quality embryos (34.6%; *p* = 0.004). Chaotic embryos comprised 8.3% of the cohort. Upon re-biopsy, none were confirmed as euploid; all remained abnormal and were reassigned to aneuploid, mosaic, or persistently chaotic categories. This finding suggests that apparent euploid results reported elsewhere may reflect technical variability and sampling limitations in PGT-A rather than accurate chromosomal normalization. *Conclusions*: The prevalence of aneuploid embryos showed a progressive increase with advancing maternal age. Chaotic embryos are heterogeneous, and re-biopsy may help refine the interpretation of complex PGT-A profiles, supporting its role as a diagnostic and quality control tool rather than a strategy to identify euploid embryos. Our study offers novel insights through age-stratified analysis, the integration of morphology with genetics in a Romanian IVF cohort, and a detailed evaluation of chaotic embryos, providing clinical recommendations for patient counseling and embryo selection.

## 1. Introduction

Embryonic euploidy represents a fundamental prerequisite for implantation and subsequent development. At the same time, aneuploidy, defined as the presence of an abnormal number of chromosomes, is the leading cause of implantation failure, early miscarriage, and congenital abnormalities [1,2]. Aneuploidy arises from errors in chromosome segregation during either mitosis or meiosis. Mitotic errors occurring in oogonia are expected to result in uniformly aneuploid oocytes and embryos, whereas mosaicism typically arises from post-zygotic mitotic errors during early embryonic divisions [3].

Mitochondria play a crucial role in maintaining oocyte quality, given their key functions in ATP production, calcium homeostasis, and regulation of apoptosis. Age-related mitochondrial dysfunction and the progressive loss of mitochondrial genome copies compromise spindle assembly, chromosomal segregation, and oocyte competence, thereby contributing to aneuploidy [4,5]. Additionally, oocyte ovulation with reduced mitochondrial integrity may occur during ovarian stimulation protocols, thereby further increasing the risk of chromosomal errors [6].

Another relevant mechanism involves the centrosome and spindle apparatus, whose disruption can impair proper segregation of homologous chromosomes [7]. Moreover, the epigenetic reprogramming that occurs during fertilization, together with the spatial nuclear organization of chromosomes, influences the fidelity of genomic transmission [8].

From a clinical perspective, preimplantation genetic testing for aneuploidy (PGT-A) has significantly improved embryo selection strategies by identifying embryos at risk of abnormal chromosomal segregation. Nevertheless, limitations remain, especially in the interpretation of mosaic and chaotic embryos. Although aneuploidy is strongly associated with poor reproductive potential, several studies have reported that mosaic embryos and, in rare cases, embryos initially classified as chaotic may result in viable pregnancies [9,10]. These findings have prompted debate about whether discarding all chaotic embryos is justified, or whether some may be artefactual results of technical or biological variation.

Therefore, understanding the distribution of aneuploidies across maternal age, identifying the prevalence of chaotic embryos, and assessing their potential for reclassification through re-biopsy are critical steps in refining embryo selection. Such knowledge has direct implications for patient counseling, clinical decision-making, and improving the overall success rates of assisted reproductive technologies.

The present study aimed to investigate the prevalence and distribution of aneuploidies across maternal age groups, with particular emphasis on the incidence of chaotic embryos and their re-biopsy outcomes. By stratifying aneuploidy patterns according to age, reporting data from a Romanian IVF cohort, and re-evaluating embryos initially classified as chaotic, the study provides new evidence with both regional relevance and practical implications for embryo selection in assisted reproduction.

## 2. Materials and Methods

### 2.1. Study Design and Setting

This retrospective observational study included embryos obtained from assisted reproduction cycles between August 2022 and July 2025. Ethical approval for the retrospective analysis of these previously collected data was granted by the Institutional Review Board of Calla IVF Center (No. 2588/A/09.05.2025). Written informed consent was obtained from all patients for the use of their embryos for genetic analysis and research. The primary outcome was the prevalence and distribution of aneuploidies across maternal age groups. Secondary outcomes included the prevalence of chaotic embryos and the re-biopsy concordance rate.

### 2.2. Study Population

A total of 230 embryos were analyzed. Patients were stratified into four maternal age groups: 25–30 years, 31–35 years, 36–40 years, and 41–50 years. Embryos included in the analysis were derived from multiple IVF cycles performed in individual patients, with more than one embryo originating from the same patient in several cases. Therefore, observations at the embryo level were not fully independent. For each age group, the number of euploid and aneuploid embryos was recorded, and the types of chromosomal abnormalities (monosomies, trisomies, complex aneuploidies, mosaicism, and chaotic profiles) were documented according to predefined classification criteria.

Patient inclusion criteria comprised women undergoing IVF with PGT-A during the study period, who had blastocyst-stage embryos available for chromosomal analysis and complete clinical and embryological data. Only embryos reaching the blastocyst stage and suitable for trophectoderm biopsy were included.

Exclusion criteria included cycles without blastocyst development, embryos not subjected to PGT-A, incomplete clinical or genetic data, embryos with inconclusive or low-quality NGS results, and cases in which re-biopsy was not technically feasible or ethically permissible.

### 2.3. Ovarian Stimulation Protocols

Controlled ovarian stimulation was performed using individualized protocols, predominantly based on a GnRH antagonist regimen, in accordance with routine clinical practice. Gonadotropins included recombinant FSH (Gonal-F, Puregon, Rekovelle, Bemfola) and hMG (Menopur, Pergoveris), with dosing adjusted according to patient age, ovarian reserve parameters, and prior response to stimulation. Ovulation was triggered using either human chorionic gonadotropin (hCG) or a GnRH agonist, depending on the individual risk of ovarian hyperstimulation syndrome, and oocyte retrieval was carried out 34–36 h later.

### 2.4. Fertilization and Embryo Culture

Fertilization was achieved through conventional IVF or intracytoplasmic sperm injection (ICSI), depending on semen parameters. Embryos were cultured using commercially available sequential culture media under standard laboratory conditions until the blastocyst stage (days 5–7). Embryo culture was performed in tri-gas incubators with controlled temperature and oxygen and carbon dioxide concentrations, in accordance with routine clinical practice. Morphological grading was performed according to the Gardner and Schoolcraft criteria, classifying embryos into good, average, or poor quality.

### 2.5. Trophectoderm Biopsy and PGT-A

Blastocysts reaching the appropriate stage of expansion underwent trophectoderm biopsy on day 5, 6, or 7. Biopsied cells were analyzed by next-generation sequencing (NGS) using the Ion ReproSeq PGS Kit (Thermo Fisher Scientific, MA, USA). Results were reported as euploid, aneuploid, or mosaic. Blastocyst quality was graded according to the Gardner and Schoolcraft criteria, with embryos categorized as good (≥3BB), average (≥3BC/3CB), or poor quality (≤3CC or equivalent). Embryos with ≥5 chromosomal abnormalities (whole or segmental) were designated as chaotic [9].

Mosaicism was defined as the presence of chromosomal abnormalities affecting 20–80% of analyzed cells, according to the laboratory’s NGS interpretation pipeline. Embryos exhibiting an abnormal cell proportion greater than 50% were classified as cases of high mosaicism.

### 2.6. Re-Biopsy of Chaotic Embryos

Chaotic embryos represent a distinct and clinically challenging subgroup in PGT-A, characterized by multiple chromosomal abnormalities and uncertain reproductive potential. Given the heterogeneity of chaotic PGT-A profiles and previous reports suggesting that some chaotic findings may reflect technical artifacts, sampling limitations, or mosaicism rather than genuine genome-wide instability, this subgroup was selected explicitly for re-biopsy. Re-biopsy was performed to assess the consistency of chromosomal findings on repeat analysis and to determine whether reclassification toward less complex karyotype profiles could occur, thereby providing diagnostic clarification rather than identifying embryos suitable for transfer.

The same NGS methodology was applied, and the results were compared with the initial PGT-A diagnosis. Although the number of re-biopsied embryos was relatively limited, this reflects the low prevalence of chaotic embryos in clinical practice and the ethical constraints regarding embryo donation for research. All embryos included in the re-biopsy analysis had been previously vitrified in accordance with standard clinical protocols. The embryos selected for re-biopsy were thawed after vitrification in accordance with standard clinical protocols, and re-biopsy was performed during subsequent clinical cycles. A new trophectoderm region, spatially distinct from the initial biopsy site, was targeted in order to minimize resampling of the same cell population. Re-biopsy samples were analyzed using the same NGS platform and standardized laboratory pipeline as the initial PGT-A assessment. Technical replicates were not routinely performed; however, strict quality control criteria and established analytical thresholds were applied to minimize amplification bias and ensure result consistency.

### 2.7. Statistical Analysis

Statistical analyses were performed using IBM SPSS Statistics version 24 (IBM Corp., Armonk, NY, USA). Categorical variables were reported as frequencies and percentages, and continuous variables as mean ± standard deviation (SD) or median and interquartile range (IQR), as appropriate. Data normality was assessed using the Shapiro–Wilk test.

Univariate comparisons were performed using the Chi-square or Fisher’s exact test for categorical variables, and Student’s *t*-test/one-way ANOVA or Mann–Whitney U/Kruskal–Wallis tests for continuous variables, as appropriate. These analyses were used for descriptive purposes.

To evaluate associations between clinical and embryological variables, multivariable regression analyses were conducted. Binary logistic regression models were applied for embryo aneuploidy (yes/no) and chaotic embryo status (yes/no), including maternal age, embryo morphological quality, and day of biopsy (when available) as independent variables. Results were reported as odds ratios (ORs) with 95% confidence intervals (95% CIs).

Linear regression was used to assess the relationships among maternal age, embryo quality, and the number of aneuploidies per embryo.

All tests were two-sided, and *p*-values < 0.05 were considered statistically significant.

## 3. Results

**Figure 1:**
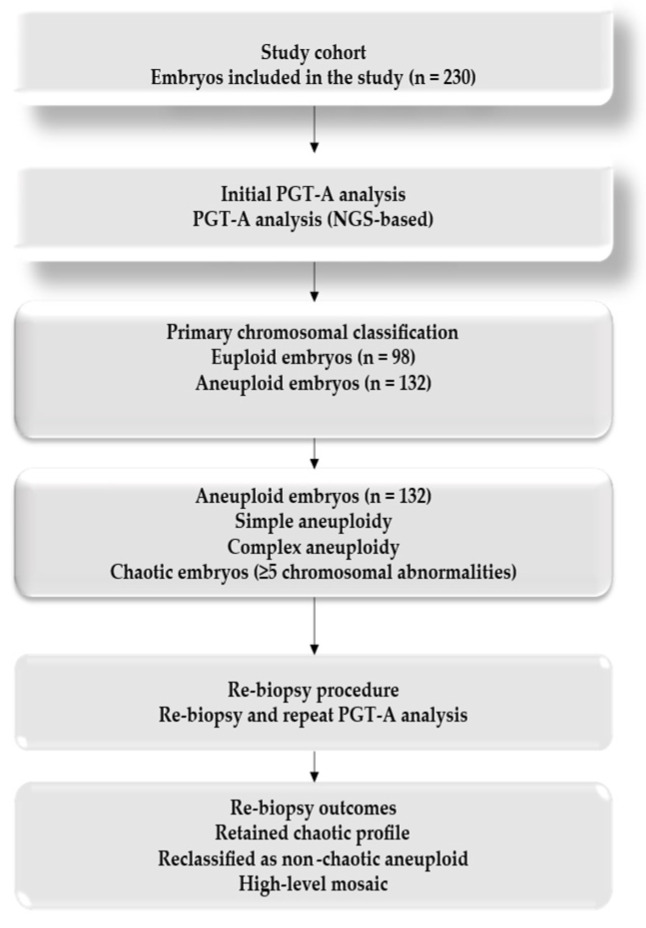
Study workflow and embryo classification process.

An overview of the study design, embryo classification, and re-biopsy workflow is presented in Figure 1.

### 3.1. Distribution of Aneuploidies by Maternal Age

A total of 230 embryos were analyzed. The prevalence of aneuploid embryos increased progressively with maternal age, ranging from 29.6% in women aged 25–30 years to 68.7% in women aged 41–50 years (*p* = 0.002, Chi-square test). The overall aneuploidy rate in the cohort was 57.4% (95% CI: 50.9–63.7%). The mean number of aneuploidies per embryo also increased significantly across age groups, from 1.4 ± 0.7 in the 25–30 years group to 3.2 ± 1.5 in the 41–50 years group (*p* < 0.001, ANOVA). Post hoc Tukey’s test confirmed significant differences between the youngest and oldest groups (*p* < 0.001), and a linear trend test demonstrated a progressive age-related increase in aneuploidy burden (*p* < 0.001). In multivariable logistic regression analysis, women aged >40 years had a significantly higher likelihood of producing aneuploid embryos compared with women aged ≤35 years (OR = 4.56, 95% CI: 2.12–9.81, *p* < 0.001), Table 1.

**Figure 2:**
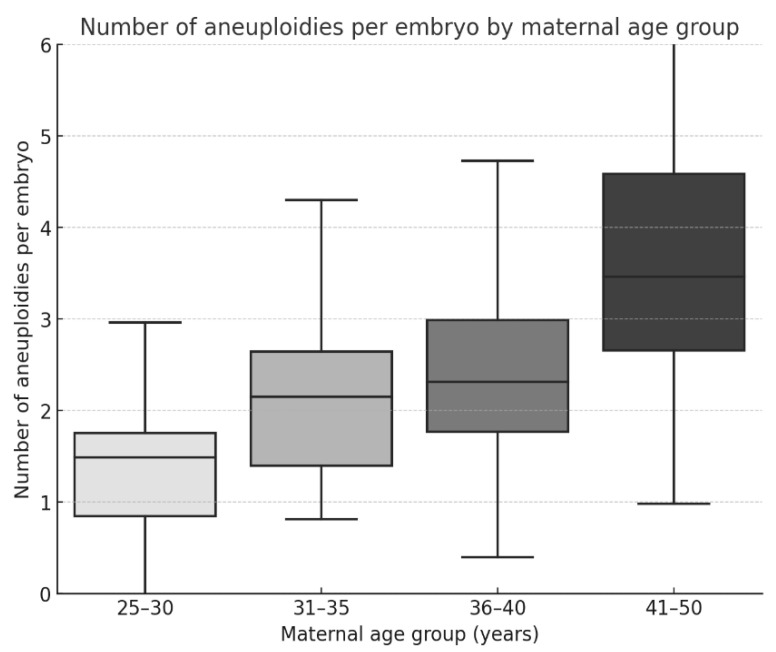
Number of aneuploidies per embryo stratified by maternal age group. Boxplots display the distribution of aneuploidy counts per embryo across age categories (25–30, 31–35, 36–40, and 41–50 years). The horizontal black line within each box represents the median, the box indicates the interquartile range (IQR), and the whiskers denote the minimum and maximum values. Different shades correspond to the respective maternal age groups.

Figure 2 illustrates the distribution of aneuploidies per embryo across the four maternal age groups. The boxplots show a clear upward shift in the median and interquartile ranges with advancing age, indicating not only a higher mean number of abnormalities but also greater variability in older patients. Outliers were predominantly observed in the 41–50 years group, which may reflect the association between advanced maternal age and both higher and more heterogeneous chromosomal instability.

**Figure 3:**
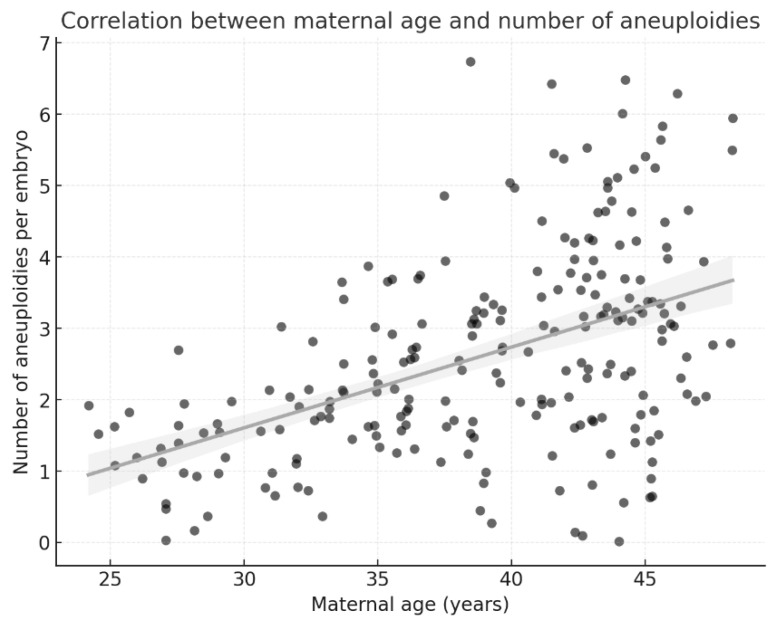
Correlation between maternal age and the number of aneuploidies per embryo. Each dot represents an individual embryo. The solid line indicates the linear regression trend, and the shaded area represents the 95% confidence interval (CI) of the regression model.

Figure 3 complements this finding by depicting the continuous relationship between maternal age and the number of aneuploidies per embryo. The scatter plot with linear regression and 95% CI confirms a strong positive correlation, highlighting maternal age as the most critical predictor of chromosomal abnormalities. Together, the two figures strengthen the statistical evidence by presenting both categorical and continuous perspectives of the same association.

### 3.2. Correlation Between Embryo Quality and Aneuploidy

Embryo morphological quality was significantly associated with chromosomal status. Aneuploidy was more prevalent in poor-quality blastocysts (72.4%) compared to average-quality (55.8%) and good-quality embryos (34.6%) (*p* = 0.004, Chi-square test), as shown in Table 2. In adjusted logistic regression analysis, poor-quality blastocysts had a significantly higher likelihood of aneuploidy than good-quality embryos (OR = 4.93, 95% CI: 2.40–10.10, *p* < 0.001), independent of maternal age.

### 3.3. Distribution of Specific Aneuploidy Types

Analysis of specific chromosomal abnormalities among the 132 aneuploid embryos revealed a heterogeneous distribution across chromosomes (Table 3). The most frequent abnormalities were trisomy 22 (11 cases, 8.3%), monosomy 16 (10 cases, 7.6%), and monosomy 22 (9 cases, 6.8%). These were followed by trisomy 19 and monosomy 21, each identified in 7 embryos (5.3%).

Less frequent abnormalities included trisomy 21 and complex aneuploidies, each observed in 6 cases (4.5%); monosomy X, observed in 5 cases (3.8%); and trisomy 16 and monosomy 15, each observed in 4 cases (3.0%). Overall, abnormalities involving chromosomes 16, 21, and 22 accounted for a substantial proportion of aneuploid findings, underscoring their susceptibility to segregation errors. The presence of complex aneuploidies further highlights the contribution of chromosomal instability within the studied cohort.

Table 3 shows that the most frequent abnormalities were trisomy 22 (8.3%), monosomy 16 (7.6%), and monosomy 22 (6.8%), followed by trisomy 19 (5.3%) and chromosome 21 abnormalities, including monosomy 21 (5.3%) and trisomy 21 (4.5%). Monosomy X accounted for 3.8% of cases, whereas complex aneuploidies accounted for 4.5%, indicating that a proportion of embryos exhibited extensive chromosomal instability, consistent with complex aneuploidy patterns.

### 3.4. Classification of Chaotic Embryos

Among the 230 embryos analyzed, 19 (8.3%) were initially classified as chaotic, according to the predefined classification criteria, Table 4. Chaotic embryos were significantly more frequent in women aged 41–50 years (13/109; 11.9%) compared with younger patients (≤40 years), in whom chaotic profiles accounted for fewer than 6% of embryos (*p* = 0.01, Chi-square test).

When stratified by embryo morphology, chaotic status was observed in 15.8% of poor-quality blastocysts, compared with 3.9% of average-quality and 1.9% of good-quality embryos (*p* < 0.01). These findings indicate a strong association between impaired embryo morphology and extreme chromosomal instability.

In adjusted logistic regression analysis, advanced maternal age remained independently associated with chaotic embryo status, with women aged 41–50 years having higher odds than women aged ≤35 years (OR = 2.8, 95% CI: 1.1–7.3, *p* = 0.03). In addition, poor-quality blastocysts exhibited significantly increased odds of being classified as chaotic compared with good-quality embryos (OR = 8.9, 95% CI: 1.1–72.0, *p* = 0.04), independent of maternal age.

**Figure 4:**
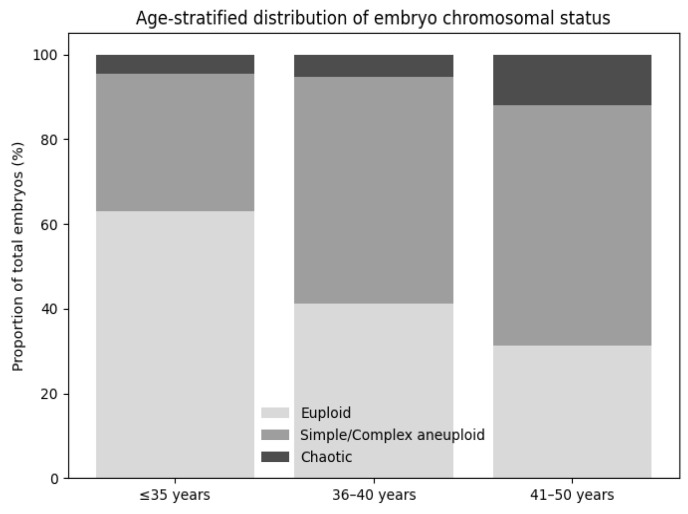
Age-stratified distribution of embryo chromosomal status.

Figure 4 shows a clear age-related shift in embryo chromosomal status when proportions are calculated relative to the total number of embryos analyzed. In women ≤35 years, euploid embryos predominate, while aneuploid embryos are mainly simple or moderately complex. With advancing maternal age, the proportion of euploid embryos decreases and is progressively replaced by more complicated and chaotic aneuploidy profiles, reaching the highest prevalence in women aged 41–50 years. These findings indicate that maternal age influences not only the frequency but also the severity of embryonic chromosomal abnormalities.

### 3.5. Re-Biopsy Outcomes of Chaotic Embryos

Importantly, none of these embryos were confirmed as euploid. Instead, the outcomes revealed different abnormal karyotypes, namely monosomy 16 (n = 1), trisomy 5 (n = 1), trisomy 22 (n = 1), complex aneuploidies involving multiple chromosomes (n = 5), persistent chaotic profiles (n = 2), and high mosaicism of chromosome 21 (n = 1), as shown in Table 5.

All remained abnormal and were reassigned to other categories, such as monosomy, trisomy, complex aneuploidies, persistent chaotic profiles, or high mosaicism. This supports the view that reports of euploid outcomes in chaotic embryos are most likely due to technical artifacts rather than accurate chromosomal normalization. This analysis was descriptive in nature and aimed to characterize the spectrum of chromosomal abnormalities observed in the cohort, without assessing predictive associations.

This re-biopsy analysis was descriptive and aimed to assess the consistency and interpretive reliability of initial PGT-A classifications rather than to evaluate predictive associations or clinical outcomes.

Taken together, these findings emphasize that no chaotic embryo can be considered genetically normal since their initial classification reflects severe chromosomal instability. The lack of euploid outcomes in our cohort contrasts with some previous reports, reinforcing that chaotic embryos should be regarded as having minimal or no reproductive potential [9].

## 4. Discussion

Our results indicate an apparent increase in the prevalence and complexity of aneuploidies with advancing maternal age, confirming that maternal age is a key determinant of chromosomal abnormalities in embryos. In our cohort, the aneuploidy rate increased from 29.6% in women aged 25–30 years to 68.7% in women aged 41–50 years. This association was further supported by multivariable logistic regression, which showed that women aged 40 years or older had a more than 4-fold increased likelihood of producing aneuploid embryos compared with women aged 35 years or younger. This finding is consistent with the large-scale study by Franasiak et al., who analyzed over 15,000 biopsies and reported an exponential increase in aneuploidy after age 35 [1]. Other reports also confirm that both numerical and structural chromosomal abnormalities accumulate with age, mainly due to meiotic errors and mitochondrial dysfunction [11,12,13].

In addition to maternal age, embryo morphology emerged as an independent predictor of chromosomal status in our regression models. Poor-quality blastocysts showed a significantly higher likelihood of aneuploidy, even after adjustment for maternal age, suggesting morphological impairment and underlying genomic instability rather than a purely subjective laboratory assessment.

These findings align with previous reports indicating that while morphology alone cannot reliably predict euploidy, it remains a clinically relevant marker when interpreted alongside PGT-A results [9,14,15].

The clinical implications are relevant for patient counseling: younger women can expect a higher proportion of euploid embryos available for transfer. In contrast, in older patients, the probability of obtaining a euploid blastocyst decreases dramatically. This emphasizes the need for age-stratified strategies in IVF, including early counseling on fertility preservation [12].

The correlation between blastocyst morphology and aneuploidy observed in our study indicates that poor-quality embryos are more likely to be aneuploid (72.4%) than good-quality embryos (34.6%). This finding aligns with the conclusions of Capalbo et al., who demonstrated that embryo grading remains a valuable predictor of chromosomal constitution, even in the era of PGT-A [9]. Similarly, Victor et al. showed that blastocysts with lower morphological scores had nearly twice the aneuploidy rate compared with high-grade embryos [14].

Nevertheless, several studies caution that morphology alone is insufficient, as euploid embryos with lower morphological quality may still implant successfully [15]. Therefore, combining morphological grading with genetic testing provides a more comprehensive assessment, allowing for better prioritization of embryos for transfer. Our data reinforce this integrative approach.

In our cohort, chaotic embryos accounted for 8.3% of all biopsied blastocysts, a prevalence comparable to the 4–10% reported in previous studies [10,16,17]. Chaotic embryos are defined by the presence of ≥5 chromosomal abnormalities and have long been considered non-viable. However, emerging evidence, including our findings, suggests that this category is heterogeneous. While extremely rare live births from embryos initially classified as chaotic have been reported in the literature [18,19,20], such cases are exceptional and likely reflect technical artifacts rather than accurate chromosomal normalization.

The prevalence of chaotic embryos observed in our cohort (8.3%) is broadly comparable to rates reported in larger international datasets, although considerable variability has been described across studies. Such differences may reflect variations in patient demographics, particularly maternal age distribution, as well as differences in laboratory protocols, including biopsy techniques, NGS platforms, and analytical thresholds used to define chaotic profiles. In addition, regional and population-specific characteristics, as well as center-specific quality control practices, may further contribute to the heterogeneity observed across studies. These factors highlight the importance of interpreting chaotic PGT-A results within the specific clinical and laboratory context in which they are generated.

Our analysis of chaotic embryos highlighted heterogeneity in re-biopsy outcomes, with some embryos retaining a chaotic profile and others being reclassified as non-chaotic aneuploid, supporting the interpretation that chaotic PGT-A profiles may reflect profound chromosomal instability rather than clearly reversible genomic alterations.

Rare cases of euploid reclassification have been reported in the literature, as highlighted by Popovic et al. and Viotti, who emphasized the diagnostic challenges associated with mosaicism and technical artifacts in PGT-A [17,18]. These observations support the hypothesis that specific chaotic profiles may also reflect methodological limitations, such as amplification bias or apoptotic cell contamination, rather than uniformly representing true chromosomal chaos.

Our re-biopsy of 19 chaotic embryos revealed that none were confirmed as euploid. Instead, several embryos were reassigned to other abnormal categories, including monosomy, trisomy, complex aneuploidies, and persistent chaotic profiles. These results are consistent with the observations of Calull et al. and Druckenmiller Cascante et al., who noted that apparent euploid reclassifications of chaotic embryos in some reports are more likely to be attributable to technical variability in next-generation sequencing rather than genuine chromosomal normalization [19,20]. Thus, our findings suggest that chaotic embryos exhibit profound chromosomal instability and may have minimal reproductive potential. Although studies such as that of Calull et al. [19] have recently addressed the prevalence and re-biopsy outcomes of chaotic embryos, our findings add complementary value in several important respects. First, this study provides data from an Eastern European IVF cohort, a population that remains underrepresented in the PGT-A literature. Second, we performed a detailed age-stratified analysis, highlighting the marked increase in chaotic embryos among women over 40 years of age, with direct implications for patient counseling and clinical decision-making. Finally, the absence of euploid reclassification after re-biopsy using a single standardized NGS platform supports a methodological interpretation of chaotic profiles as reflecting profound chromosomal instability, reinforcing the role of re-biopsy as a quality control tool rather than a rescue approach. The variability observed across chaotic embryos is clinically meaningful. While some embryos retained a chaotic profile at re-biopsy and others were reclassified as non-chaotic aneuploid, these findings highlight the heterogeneous nature of chaotic PGT-A profiles and their limited reproductive relevance, in contrast with isolated reports in the literature. This variability may reflect biological heterogeneity and technical limitations of PGT-A, rather than evidence of distinct subcategories. Similar findings were reported by Rodrigo et al., who suggested that chaotic embryos may not represent a uniform biological entity but rather a diagnostic spectrum influenced by methodological constraints [16].

Our findings support a cautious interpretation of chaotic PGT-A results. Traditionally, embryos with chaotic profiles were automatically excluded from transfer. Although a small proportion were reclassified as euploid after re-biopsy, these cases may represent diagnostic artifacts rather than accurate chromosomal normalization. Our results, combined with international evidence, suggest that re-biopsy may provide additional diagnostic clarity, but chaotic embryos should continue to be regarded as having minimal or no reproductive potential.

From a clinical perspective, the absence of euploid reclassification after re-biopsy in our cohort supports a cautious interpretation of chaotic PGT-A results. Rather than indicating transient or reversible chromosomal alterations, chaotic profiles appear to reflect profound genomic instability, reinforcing the role of re-biopsy primarily as a quality control and diagnostic clarification tool rather than a rescue strategy. The higher prevalence of chaotic embryos with advancing maternal age further highlights the importance of age-stratified counseling when discussing PGT-A findings with patients.

Although no euploid embryos were identified at re-biopsy in our cohort, larger datasets may capture rare cases with euploid potential. In addition, it should be considered that some embryos classified as aneuploid based on trophectoderm biopsy may harbor mosaicism confined to the trophectoderm rather than the inner cell mass, underscoring the need for cautious interpretation of PGT-A results. Given the relatively small number of chaotic embryos, regression analyses involving this outcome were considered exploratory. The resulting effect estimates may show reduced precision, as reflected by wide confidence intervals, and should be interpreted cautiously.

Overall, integrating multivariable regression analyses with descriptive statistics strengthens the statistical foundation of our findings and supports the interpretation of observed associations, given the limitations of a retrospective design.

A key limitation of this study is that multiple embryos originated from the same patient, and therefore, observations at the embryo level were not fully independent. Although regression models were adjusted for relevant covariates, clustered or GEE-based approaches were not applied; consequently, residual intra-patient correlation may have influenced variance estimation and regression inference.

## 5. Conclusions

This study indicates that the prevalence and complexity of aneuploidy increase with maternal age and that embryo morphology is significantly associated with chromosomal status. Although re-biopsy frequently resulted in reclassification toward less complex karyotypes, this reflected diagnostic clarification rather than biological normalization, as no euploid embryos were identified in our cohort. Embryos classified as chaotic were predominantly characterized by profound chromosomal instability, and re-biopsy frequently resulted in alternative aneuploid classifications, which may reflect extensive genomic disruption rather than reversible chromosomal alterations. Clinically, these findings highlight the importance of age-related patient counseling and the integration of morphological assessment with genetic testing. At the same time, they support a cautious interpretation of chaotic PGT-A results and underscore the need for standardized laboratory and clinical protocols, as well as larger, multicenter prospective studies, to better define the reproductive potential and optimal management of chaotic embryos in assisted reproduction.

## Figures and Tables

**Table 1:** Distribution of euploid and aneuploid embryos by maternal age group.

Age Group (Years)	Total Embryos	Euploid n (%) [95% CI]	Aneuploid n (%) [95% CI]	Mean Aneuploidies ± SD
25–30	27	19 (70.4) [53.2–84.1]	8 (29.6) [15.9–46.9]	1.4 ± 0.7
31–35	38	22 (57.9) [40.8–73.7]	16 (42.1) [26.3–59.2]	1.9 ± 0.8
36–40	56	23 (41.1) [28.1–55.0]	33 (58.9) [44.9–71.8]	2.5 ± 1.1
41–50	109	34 (31.3) [22.5–41.2]	75 (68.7) [59.3–76.8]	3.2 ± 1.5
Total	230	98 (42.6) [36.3–49.0]	132 (57.4) [50.9–63.7]	

**Table 2:** Distribution of euploid and aneuploid embryos according to embryo quality.

Embryo Quality	Total Embryos	Euploid *n* (%)	Aneuploid *n* (%)
Good	52	34 (65.4%)	18 (34.6%)
Average	77	36 (46.8%)	41 (53.2%)
Poor	101	28 (27.7%)	73 (72.4%)
Total	230	98 (42.6%)	132 (57.4%)

**Table 3:** Most frequent aneuploidy types observed in the cohort.

Aneuploidy Type	Cases (n)	% of Aneuploid Embryos (*n* = 132)
Trisomy 22	11	8.3%
Monosomy 16	10	7.6%
Monosomy 22	9	6.8%
Trisomy 19	7	5.3%
Monosomy 21	7	5.3%
Trisomy 21	6	4.5%
Complex aneuploid	6	4.5%
Monosomy X	5	3.8%
Trisomy 16	4	3.0%
Monosomy 15	4	3.0%

Note: Each aneuploid embryo was assigned a single, mutually exclusive category based on the predominant chromosomal abnormality identified. Percentages were calculated using the total number of aneuploid embryos (*n* = 132) as the denominator.

**Table 4:** Distribution of chaotic embryos according to maternal age and embryo quality.

Group	Total Embryos	Chaotic Embryos *n* (%) [95% CI]
Age ≤ 35 years	65	3 (4.6) [1.6–12.7]
Age 36–40 years	56	3 (5.4) [1.9–14.6]
Age 41–50 years	109	13 (11.9) [7.1–19.2]
Total by age	230	19 (8.3) [5.4–12.7]
Good-quality embryos	52	1 (1.9) [0.3–9.9]
Average-quality embryos	77	3 (3.9) [1.3–11.0]
Poor-quality embryos	101	15 (14.9) [9.2–23.3]
Total by quality	230	19 (8.3) [5.4–12.7]

**Table 5:** Results of re-biopsy in embryos initially diagnosed as chaotic.

Outcome Type	Cases (n)	%
Monosomy 16	1	5.3%
Trisomy 5	1	5.3%
Trisomy 22	1	5.3%
Complex aneuploidies	5	26.3%
Persisting chaotic profile	2	10.5%
High mosaicism (XXI)	1	5.3%
Other aneuploid outcomes	8	42.1%

Note: Each embryo was assigned a single, mutually exclusive classification based on the predominant chromosomal abnormality identified at re-biopsy. No embryo was counted in overlapping categories. A detailed breakdown of the category “Other aneuploid outcomes” is provided in Supplementary Table S1.

## Data Availability

The data presented in this study are available from the corresponding author upon reasonable request due to ethical and privacy restrictions related to patient data.

## Supplementary Materials

The following supporting information can be downloaded at https://www.mdpi.com/article/10.3390/medicina62020247/s1, Table S1: Breakdown of “Other aneuploid outcomes” after re-biopsy.
