# Supplementary material for: Aneuploidy Patterns and Chaotic Embryos in IVF: Age-Stratified Analysis and Re-Biopsy Outcomes from a Romanian Cohort
Source: Medicina (Kaunas). 2026 Jan 24;62(2):247. doi: 10.3390/medicina62020247 (PMC12941486; doi:10.3390/medicina62020247)
Supplement: Supplementary file 1 [file medicina-62-00247-s001.zip › medicina-4098764-supplementary.pdf]

Supplementary Materials

Table S1. Breakdown of "Other aneuploid outcomes" after re-biopsy

Subtype of aneuploid outcome (re-biopsy)	n
Segmental aneuploidy involving one or more chromosomes	2
Combined monosomy and trisomy (non-chaotic)	3
Multiple non-chaotic aneuploidies (≤4 abnormalities)	3
Total ("Other aneuploid outcomes")	8
